# Dr. Bernard Baehr

**Published:** 1885-01

**Authors:** 


### DR. BERNARD BAEHR.

There died at Gmunden, on the 21st of October, 1884, at the residence of his host, the Duke of Cumberland, a very notable homœopathic physician—Dr. Bernard Baehr, of Hanover.

Baehr was born at Hanover on the 17th of April, 1828, and studied at the Universities of Göttingen and Vienna. He already became acquainted with Homœopathy while in Vienna, and in 1855 he brought himself prominently to the fore by the publication of his monograph on Digitalis, to which the Homœopathische Central Verein of Germany awarded its prize; and then he brought out in 1862 his well-known work on therapeutics, *Therapie nach den Grundsatzen der Homoopathie*, which procured him a considerable reputation in this country and in America. Our lamented colleague died of tuberculosis consecutive to diabetes.

We quite agree with Dr. Metz, from whose obituary notice in the *Allgemeine Homœopathische Zeitung* we borrow these particulars, that Homœopathy loses an able and faithful representative in Dr. Baehr. May the many blessings he has conferred on others now return to those of his own house who survive and bemourn him.—*Homœopathic World.*

